# Research progress on antitumor effects of sea buckthorn, a traditional Chinese medicine homologous to food and medicine

**DOI:** 10.3389/fnut.2024.1430768

**Published:** 2024-07-09

**Authors:** Duojie Xu, Ling Yuan, Fandi Meng, Doudou Lu, Mengying Che, Yating Yang, Wenjing Liu, Yi Nan

**Affiliations:** ^1^Traditional Chinese Medicine College, Ningxia Medical University, Yinchuan, Ningxia, China; ^2^College of Pharmacy, Ningxia Medical University, Yinchuan, Ningxia, China; ^3^School of Clinical Medicine, Ningxia Medical University, Yinchuan, Ningxia, China; ^4^Key Laboratory of Ningxia Minority Medicine Modernization Ministry of Education, Ningxia Medical University, Yinchuan, Ningxia, China

**Keywords:** sea buckthorn, *Hippophae Fructus*, tumor, cancer, homology of medicine and food

## Abstract

Sea buckthorn (*Hippophae Fructus*), as a homologous species of medicine and food, is widely used by Mongolians and Tibetans for its anti-tumor, antioxidant and liver-protecting properties. In this review, the excellent anti-tumor effect of sea buckthorn was first found through network pharmacology, and its active components such as isorhamnetin, quercetin, gallic acid and protocatechuic acid were found to have significant anti-tumor effects. The research progress and application prospect of sea buckthorn and its active components in anti-tumor types, mechanism of action, liver protection, anti-radiation and toxicology were reviewed, providing theoretical basis for the development of sea buckthorn products in the field of anti-tumor research and clinical application.

## 1 Introduction

Sea buckthorn, a dry and mature fruit of the *Elaeagnaceae* family, is widely used as a medicinal material by Mongolian and Tibetan people. This deciduous shrub or tree is characterized by its numerous thorns and lanceolate leaves, which are often arranged in opposite pairs. The upper surface of the leaves is green and covered with fine hairs, while the lower surface appears silvery white due to shield-shaped scales. The spherical fruits of sea buckthorn have an orange-yellow or brown-red exterior and measure 4–8 mm in diameter. Its flesh is soft, glossy, and contains black oval seeds. Harvesting usually takes place when the fruit ripens from August to October or when it becomes frozen solid (1). In 1971, Arne Rousi, a Finnish botanist, conducted an extensive study on sea buckthorn and classified it into nine subspecies. Among these subspecies, five can be found in China. Sea buckthorn possesses various traits such as light tolerance, heat resistance, cold hardiness, and drought adaptability which make it an ideal plant for ecological greening purposes. Furthermore, sea buckthorn serves both medicinal and culinary purposes making it highly versatile with great potential for application. According to the Chinese Pharmacopoeia (1) sea buckthorn has a sour taste combined with astringency; it invigorates the spleen function while eliminating food stagnation; relieves coughs by removing phlegm; promotes blood circulation; disperses blood stasis; treats conditions like spleen deficiency accompanied by abdominal pain due to food accumulation; coughs with excessive phlegm production along with chest congestion causing heartache; menstrual disorders caused by blood stasis accumulation; as well as injuries resulting in hematoma formation leading to pus accumulation alongside swelling. Recent pharmacological research has demonstrated that sea buckthorn possesses noteworthy therapeutic properties in the management of cardiovascular diseases, anti-tumor, anti-oxidation, and liver protection. Importantly, it should be emphasized that the medicinal benefits associated with sea buckthorn extend beyond its fruit alone. The medicinal value inherent in its leaves, oil, and seeds.

Cancer, also referred to as malignant tumors, is characterized by aberrant mutations in normal cells that undergo uncontrolled and excessive proliferation, eventually leading to metastasis. According to the latest report in 2020, there were approximately 19.29 million new cases of malignant tumors worldwide, with a staggering 9.96 million deaths attributed to this disease. Furthermore, it is projected that by 2040, there will be an estimated 28.4 million new cancer cases globally (2). As of July 2019, China’s tumor registry encompassed a population of around 438 million individuals, accounting for approximately 31.5% of the country’s total populace. Over the past four decades, China has witnessed a significant surge in the burden of cancer; thus highlighting the urgent need to address this ailment as one of the most critical public health challenges faced in the twenty-first century (3). Currently, the management of malignant tumors primarily encompasses surgical resection, chemoradiotherapy, photothermal therapy, gene therapy, immunotherapy, and other modalities (4). The treatment of cancer is contingent upon its stage of progression; early detection leads to improved therapeutic outcomes and prolonged survival. In the initial phases of cancer development, lesions can be surgically excised to achieve maximal radical intervention. However, a majority of patients are diagnosed during intermediate or advanced stages when treatment becomes challenging.

**Figure 1:**
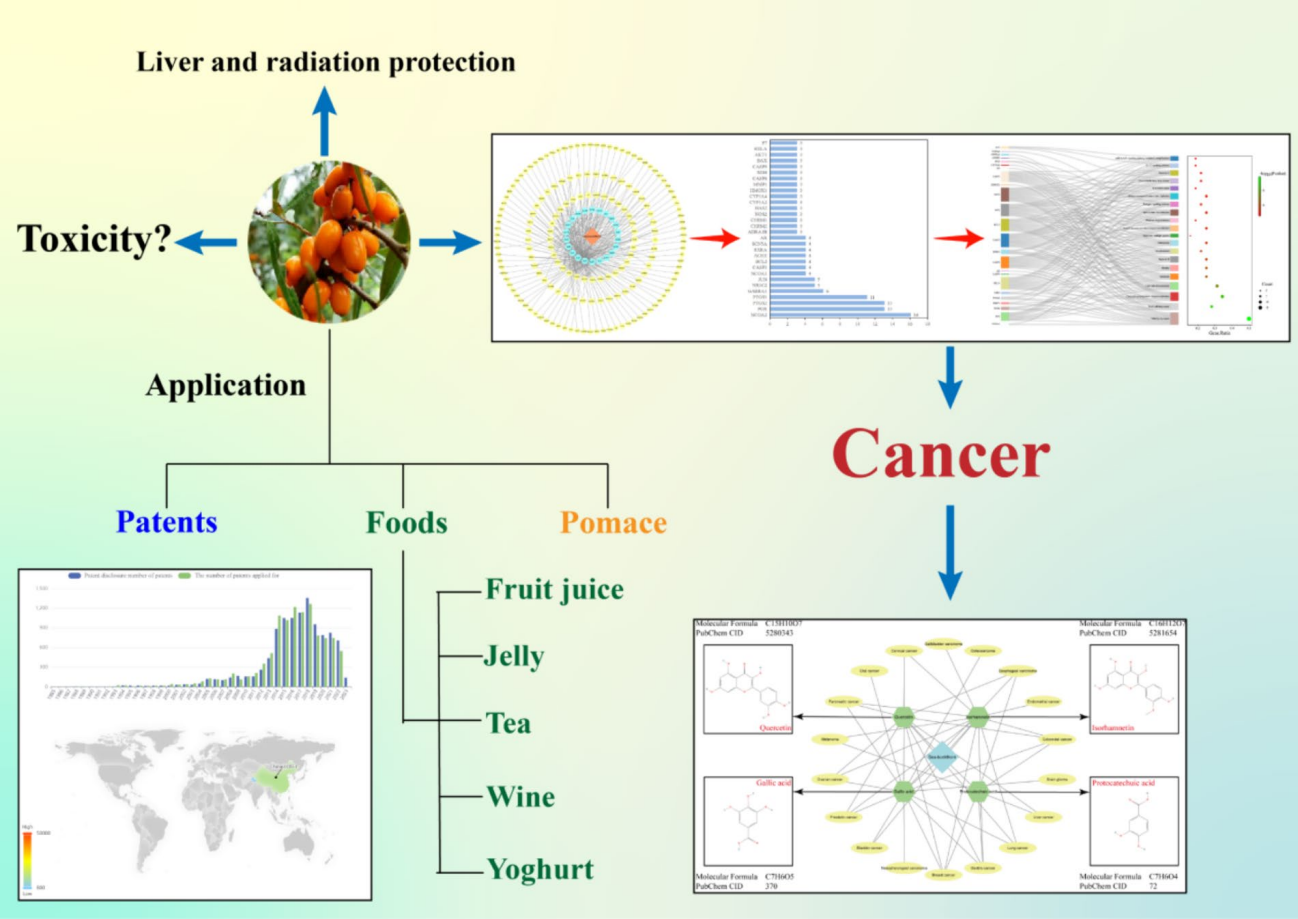
Flow chart.

Due to the global prevalence of diet-related chronic diseases, the concept of Food is Medicine was proposed by Downer et al. (5), highlighting its potential for managing and treating patients with chronic illnesses. Chinese medicine has long embraced the belief that “medicine and food have the same origin,” as evident in ancient texts like Huangdi Neijing, which states that consuming food on an empty stomach serves as nourishment while being medicinal when consumed by patients. Recognizing this synergy, the National Health and Medical Commission has identified a total of 110 traditional Chinese medicines that possess both nutritional and medicinal properties, including sea buckthorn, with ongoing efforts to expand this list further. Traditional Chinese medicine (TCM) plays a pivotal role in cancer prevention and treatment (6). The treatment of cancer with TCM primarily serves as adjuvant therapy. By modulating the internal environment of the body, it can effectively impede tumor growth and reduce metastasis. Additionally, TCM has the potential to regulate immunity and alleviate patients’ discomfort and adverse reactions during radiotherapy and chemotherapy (7). In this review, we comprehensively examine the anti-tumor mechanisms of key active compounds found in sea buckthorn. Furthermore, we investigate the protective effects of sea buckthorn on liver function and radiation-induced damage. Considering its dual role as both medicine and food source, we also explore the toxicity profile and applications of sea buckthorn. Please refer to Figure 1 for a visual representation of our research flowchart.

## 2 Screening of sea buckthorn related pathways

**Figure 2:**
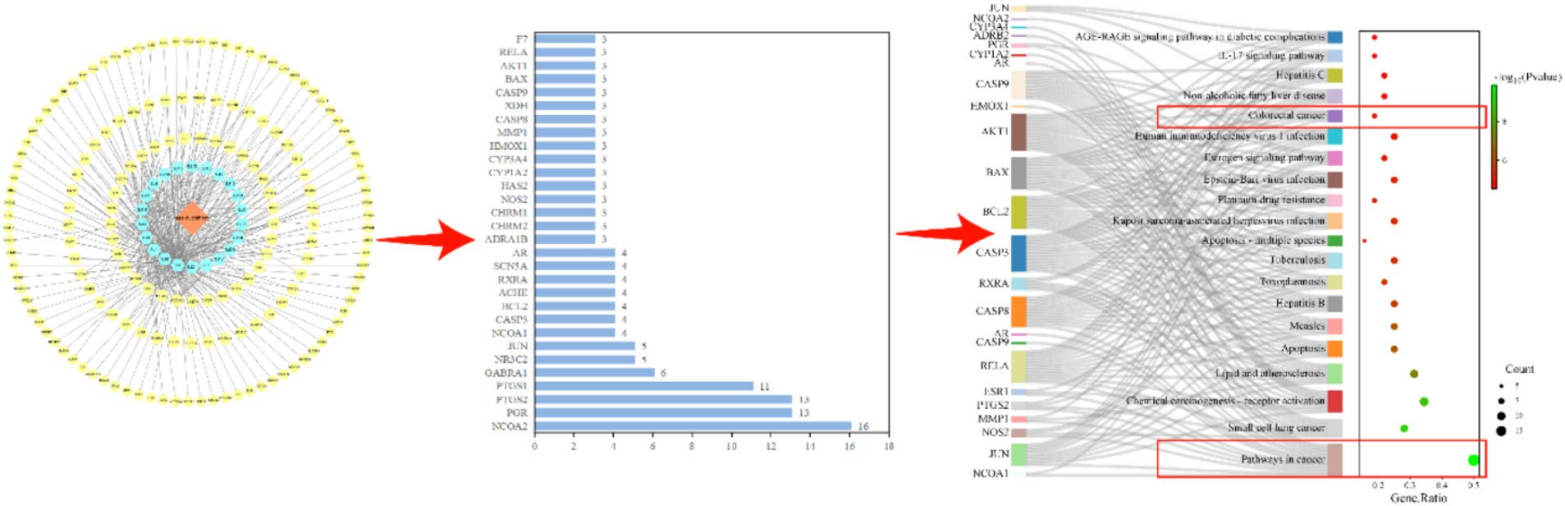
Screening of related targets and pathways of sea buckthorn.

**Figure 3:**
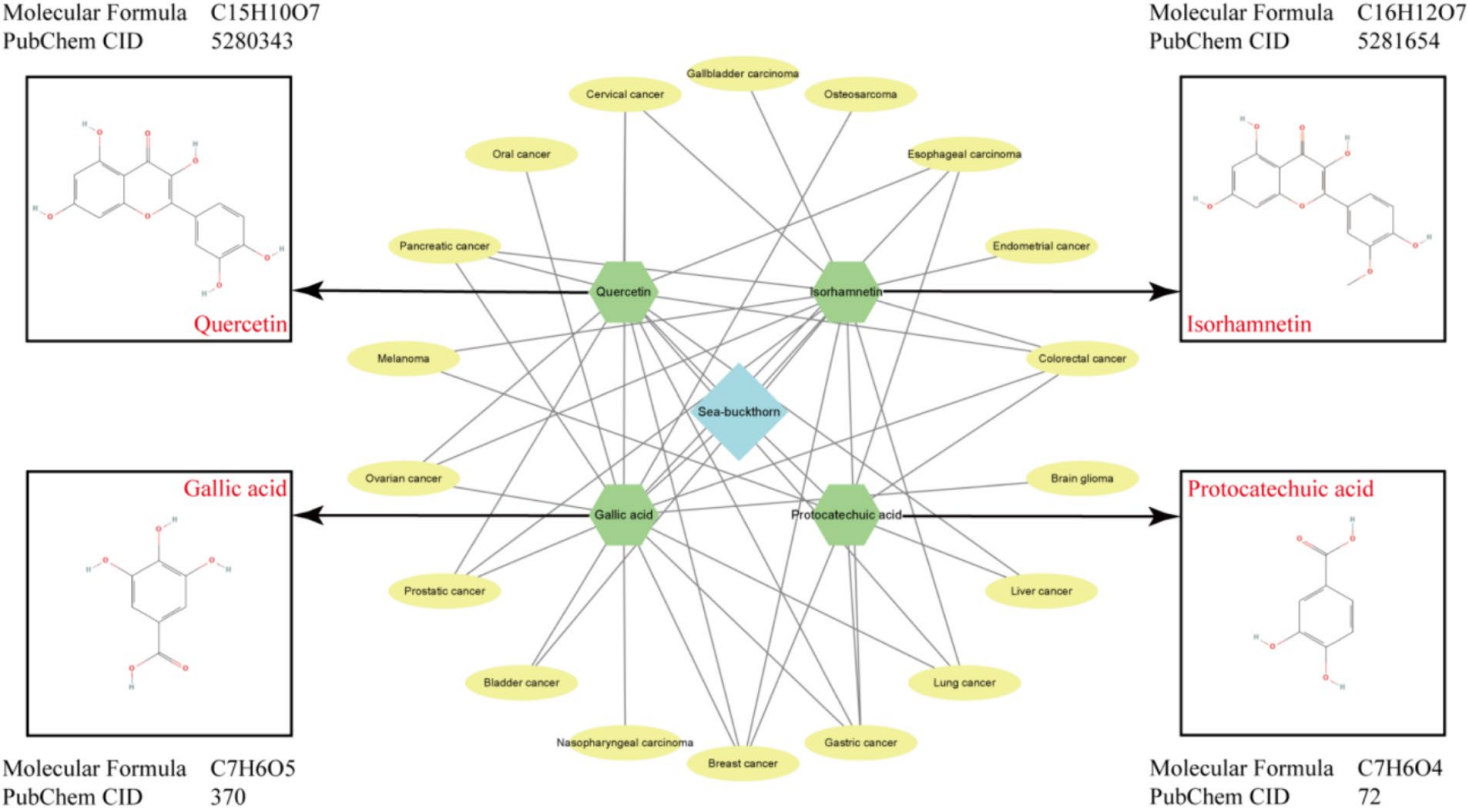
Main active components, structural formulas and types of cancer treated by sea buckthorn.

We employed bioinformatics methods, utilizing the TCMSP^1^ and DAVID^2^ online databases, as well as Cytoscape3.9.1 software and the bioinformatics online platform^3^, to conduct enrichment analysis of the active components of sea buckthorn and their targets (Figure 2; Table 1). The findings reveal that a majority of genes are enriched in cancer-related pathways, including Small cell lung cancer and Colorectal cancer. Consequently, our focus is directed towards investigating the effects of sea buckthorn on tumors for this review (Figure 3).

**Table 1:** Active ingredients of sea buckthorn and their ID.

ID	Ingredient
SJ1	ent-Epicatechin
SJ2	quercetin
SJ3	isorhamnetin
SJ4	beta-sitosterol
SJ5	sitosterol
SJ6	kaempferol
SJ7	FA
SJ8	Stigmasterol
SJ9	(+)-catechin
SJ10	CLR
SJ11	pelargonidin
SJ12	ZINC04073977
SJ13	Mandenol
SJ14	24-epicampesterol
SJ15	LAN
SJ16	rhein
SJ17	(3S,5R,10S,13R,14R,17R)-17-(1R)-1,5-dimethyl-4-methylenehexyl-4,4,10,13,14-pentamethyl-2,3,5,6,7,11,12,15,16,17-decahydro-1H-cyclopentaaphenanthren-3-ol
SJ18	beta-carotene
SJ19	5,7-dihydroxy-2-(3-hydroxy-4-methoxyphenyl)chroman-4-one
SJ20	Schottenol
SJ21	14-methyl-alpha-sitosterol
SJ22	ergostenol

## 3 Main active ingredients and pharmacological effects

### 3.1 Flavonoid

Flavonoids are generally considered to be the primary active ingredients in sea buckthorn, sea buckthorn varieties in different component content determination of total flavonoids in different parts of the following shows that the highest flavonoid in the sea buckthorn, accounted for 76%, fruit with 14% times, minimum content of seed, which is about 10% (8). Currently, over 50 flavonoids have been identified from sea buckthorn fruit, including quercetin, isorhamnetin, kaempferol, and other flavonoid aglycones. Additionally, glucose, rhamnose-rutin, and other sugar groups combine to form flavonol glycosides. Among these compounds, isorhamnetin derivatives account for 65% of total flavonols, while quercetin derivatives make up 25%. Isorhamnetin is a natural small molecule flavonoid also known as 3,5,7-trihydroxy-2-(4-hydroxy-3-methoxyphenyl)benzopyran-4-one (9). Relevant studies have revealed notable variations in the types and compositions of flavonoids among different subspecies, varieties, and origins of sea buckthorn. Quercetin is commonly present in the flowers, leaves, and fruits of numerous plants primarily as glycosides. It exhibits pharmacological properties such as antioxidation, anti-inflammatory effects, hypoglycemic activity, anticancer potential, as well as prevention and treatment capabilities for cardiovascular and cerebrovascular diseases (10). In the process of extracting flavonoids from sea buckthorn, the flavonoid content obtained by different extraction methods was also different. The flavonoid content obtained by ultrasonic and microwave extraction methods was about 22 mg/g, and only 12 mg/g obtained by aqueous two-phase extraction method. However, the microwave extraction method takes less time, but the ultrasonic and aqueous two-phase extraction methods have higher safety (11).

### 3.2 Polyphenols

Sea buckthorn is rich in over 30 polyphenolic compounds, total polyphenol content of sea buckthorn leaves is about 3 to 4 times of sea buckthorn fruit (12), predominantly gallic acid and protocatechuic acid, as well as p-hydroxybenzoic acid, vanillic acid, and salicylic acid, among others. Notably, gallic acid exhibits antibacterial, antiviral, and antitumor properties (13). The abundance of polyphenolic compounds in sea buckthorn contributes significantly to its role in cardiovascular protection. Protocatechuic acid or 3,4-dihydroxybenzoic acid serves as the primary metabolite of anthocyanins and possesses antioxidant, antibacterial, anti-inflammatory, and anti-tumor effects (14).

### 3.3 Fatty acids

The oil content of Sea buckthorn in Central Asia reaches an impressive 22.57%, while in China it ranges from 2.38 to 12.07%. Sea buckthorn fruit oil is rich in fatty acids, with significant variations observed among different varieties and origins of sea buckthorn, among them, the fatty acid content of sea buckthorn fruit is about 5%, and that of seed is about 70% (15). The predominant fatty acids found in sea buckthorn are unsaturated, including palmitoleic acid, palmitic acid, oleic acid, linoleic acid, and linolenic acid. Notably, the content of palmitoleic acid can be as high as 32 to 53%. Relevant research has demonstrated that palmitoleic acid exhibits potential for preventing, controlling, and improving chronic metabolic diseases and inflammation (16). In the extraction process of sea buckthorn fruit oil, the use of organic solvent extraction oil rate of up to 22–28%, but its security is low; Squeeze the extraction operation is simple, but the oil rate less than 1%; Enzymatic, supercritical CO_2_ extraction, and ultrasonic assisted enzymatic oil rate between 2 and 6% (17).

### 3.4 Other

Sea buckthorn is enriched with bioactive compounds including triterpenoids, steroids, alkaloids, and β-carotene. Furthermore, its pharmacological potential against tumor growth has been substantiated through pertinent research studies.

## 4 Antitumor effect

### 4.1 Isorhamnetin

The anti-tumor potential of isorhamnetin has garnered significant attention in recent years, demonstrating a comprehensive range of anti-tumor activities, including the inhibition of cell proliferation and migration and the induction of cell apoptosis (Table 2). Notably, treatment with isorhamnetin severely disrupted the morphology of AGS-1 and HGC-27 cells. Furthermore, joint staining analysis using Caspase-3 and Annexin V revealed that the activation of apoptosis induced by isorhamnetin primarily relied on Caspase-3 activation. Importantly, subsequent CCK-8, transwell, and wound healing assays confirmed that isorhamnetin also effectively inhibited gastric cancer cell proliferation and migration (18). In HT-29 colon cancer cells, isorhamnetin’s chemoprotective properties against colon cancer are attributed to its anti-inflammatory activity as well as its inhibition of Src-mediated carcinogenesis, leading to the subsequent loss of nuclear beta catenin that relies on CSK expression (19). Furthermore, studies conducted on GBC-SD and NOZ cell lines demonstrated that isorhamnetin effectively suppressed cell proliferation and metastasis in gallbladder cancer by deactivating the PI3K/AKT signaling cascade. Additionally, it induced apoptosis while blocking the G2/M phase progression in GBC cells (20). Isorhamnetin was found to decrease the phosphorylation levels of MEK and ERK in the Ras/MAPK pathway of PANC-1 cells, leading to a significant inhibition of cell growth through S phase block. Additionally, wound healing experiments demonstrated that isorhamnetin significantly reduced the migration ability of PANC-1 cells (21). Furthermore, isorhamnetin exhibited inhibitory effects on breast cancer cell proliferation by down-regulating MMP2 and MMP9 protein expression levels. Notably, overexpression of *ESR1* promoted breast cancer cell proliferation, migration, and invasion; however, these results were reversed upon knocking down *ESR1*. The observed inhibitory effect of isorhamnetin on breast cancer cells was attributed to its ability to suppress *ESR1* gene expression (22). In the intervention of prostate cancer cells, isorhamnetin exhibits its potential by promoting apoptosis through downregulating the expression of anti-apoptotic protein Bcl-2 and upregulating the levels of pro-apoptotic proteins Bax and cytochrome C. Additionally, it plays a crucial role in suppressing metastasis by enhancing e-cadherin expression while reducing vimentin and N-cadherin expressions, as well as MMP2 and MMP9 activities. Furthermore, evaluation of the PI3K/AKT/mTOR pathway confirms that isorhamnetin effectively inhibits this signaling cascade, thereby exerting anticancer effects (23). Moreover, isorhamnetin induces G2/M phase arrest via binding to Cdk1 and inhibiting its activity through both endogenous and exogenous pathways. It also upregulates Fas, FasL, and Bax protein levels while downregulating anti-apoptotic protein Bcl-2 expression to induce apoptosis in bladder cancer cells, ultimately restraining their proliferation (24). In a breast cancer study, isorhamnetin was found to exert its effects through the inhibition of Akt/mTOR and MEK/ERK signaling pathways, thereby promoting apoptosis and inhibiting cell proliferation (25). In the investigation conducted by Luo et al. (26), it was demonstrated that isorhamnetin effectively blocks the Akt/ERK1/2 signaling pathway, leading to the inhibition of epithelial-mesenchymal transition (EMT) and subsequent suppression of lung cancer cell metastasis. Additionally, Ye et al. (27) also reported that isorhamnetin facilitates cell apoptosis by inducing endoplasmic reticulum stress via both endogenous mitochondrial apoptotic pathways and exogenous death receptors. Furthermore, this compound exhibits an ability to regulate MMP2 and MMP9 protein levels, thus impeding cell metastasis. The expression levels of Bax and Caspase-3 were upregulated, while the expression level of Bcl-2 was downregulated upon isorhamnetin intervention in the mouse skin melanoma cell line B16F10. These findings provide evidence for the pro-apoptotic ability of isorhamnetin through the inhibition of PI3K/Akt and NF-κB signaling pathways, with its inhibitory effect being associated with PFKFB4 (28). Furthermore, Juan Wei et al.’s study on cervical cancer cells demonstrated that isorhamnetin effectively hindered cell cycle progression at the initial G2/M phase by suppressing protein expressions of cyclin B1, cell division cycle 25C (Cdc25C), and Cdc2 (29).

**Table 2:** Types and mechanisms of cancer treatment with isorhamnetin.

Ingredient	Cancer	Mechanism	Phenotype	References
Isorhamnetin	Gastric cancer	The up-regulation of cytochrome c, Bax/Bcl-2 and cytochrome c in cytoplasm, and cleaved-Caspase3 and PARP lead to the imbalance of mitochondrial homeostasis and apoptosis.	Apoptosis	(18)
	Colorectal cancer	The expression of CSK is induced to inhibit oncogenic Src activity and β-catenin nuclear translocation.	Inflammation	(19)
	Gallbladder carcinoma	Block G2/M phase; up-regulate the expression of p53 and down-regulate the expression of p-PI3K and p-AKT.	Apoptosis Metastasis Proliferation	(20)
	Pancreatic cancer	Inhibit the activity of Ras/MAPK pathway; S-phase block; inhibit cell migration.	Apoptosis Migration	(21)
	Ovarian cancer	Inhibit cell viability, proliferation and invasion; down-regulate the expression of MMP2 and MMP9 proteins.	Proliferation Migration Invasion	(22)
	Prostatic cancer	Promote cell apoptosis; inhibit cell migration and invasion; the activation of PI3K/Akt/mTOR pathway is inhibited, and the expression of downstream regulatory factors of apoptosis is changed.	Apoptosis Migration Invasion	(23)
	Bladder cancer	Inhibit cell activity; induce cell G2/M phase arrest and apoptosis; increase ROS production and decrease ATP content; decrease mitochondrial membrane potential and activate AMPK signaling pathway.	Proliferation Apoptosis	(24)
	Breast cancer	Inhibit the proliferation of tumor cells and induce apoptosis; the levels of p-Akt, p-mTOR, p-MEK1/2, p-ERK1/2, Bcl-2 and Bcl-xL are down-regulated, and the level of cleaved-Caspase 3 is up-regulated.	Proliferation Apoptosis	(25)
	Lung cancer	Inhibit cell proliferation, adhesion, migration and invasion; inhibit MMP-2 and MMP-9 enzyme activities; inhibit the expression of EMT markers.	Proliferation Migration Invasion	(26)
	Endometrial cancer	Inhibit cell proliferation and metastasis; cell cycle arrest is in G2/M phase; promote cell apoptosis; raise the ROS level.	Apoptosis Proliferation Migration Invasion	(27)
	Melanoma	Inhibit cell proliferation and migration; induce cell apoptosis.	Proliferation Migration Apoptosis	(28)
	Cervical cancer	Inhibit cell proliferation; cell cycle stagnate in G2/M phase; up-regulate the expression of phosphorylated Chk2 (Thr68); down-regulate the expression of Cdc25C, Cdc2 and cyclin B1.	Proliferation	(29)

### 4.2 Quercetin

Quercetin, a flavonoid compound, exhibits anti-tumor, anti-inflammatory, analgesic properties and exerts protective effects on the cardiovascular and cerebrovascular systems (Table 3). Pertinent evidence demonstrates that treatment with quercetin in HT-29 cells results in growth inhibition, alterations in cell morphology, and induction of apoptosis (30). In liver cancer cells SMMC7721 and HepG2, quercetin activates autophagy by inhibiting the AKT/mTOR pathway while activating the MAPK signaling pathway. Consequently, this leads to the suppression of cell proliferation and initiation of apoptosis (31). Quercetin exhibits its anti-proliferative effects on pancreatic cancer cells by down-regulating c-Myc expression and suppressing EMT levels through the reduction of TGF-β1. Furthermore, it effectively hinders cell migration and invasion (32). In the investigation involving AGS cells, quercetin induces apoptosis in AGS cells via activation of the MAPK signaling pathway and modulation of TRPM7 channel activity (33). Notably, when studying the intervention of quercetin on esophageal cancer cells, it significantly impedes human esophageal cancer Eca109 cell proliferation in a time- and dose-dependent manner while concurrently inducing their apoptosis (34). Ren et al. (35) demonstrated that quercetin exhibits inhibitory effects on the proliferation of ovarian cancer cells, impedes cell cycle progression from G0/G1 to G2/M phase, and induces apoptosis *in vitro*. Ward et al. (36) discovered that quercetin effectively triggers apoptosis and secondary necrosis in three distinct types of prostate cancer cells. Further investigations revealed that the anti-prostate cancer efficacy of quercetin is mediated through its regulation of ROS, Akt, and NF-κB pathways. Lee et al. (37) demonstrated that quercetin can activate AMPK through the generation of ROS in breast cancer cells, leading to the inhibition of COX-2 expression and thereby exerting its antiproliferative and pro-apoptotic effects. Subsequently, re-treatment resulted in cell cycle arrest at the sub-G1 phase, upregulation of apoptosis-related genes, and downregulation of the survival gene VEGF. Moreover, quercetin was found to enhance the expression levels of LC3-II and beclin 1 while inhibiting p62 expression. It also increased SIRT1 protein level and pAMPK-AMPK ratio, ultimately inducing mitochondria-dependent apoptosis and autophagy while suppressing cell viability (38). Finally, in the investigation of HeLa cervical cancer cells, following intervention, genes implicated in the G2/M phase of the cell cycle (*CCNB1*, *CCNB2*, and *CDK2*), relevant genes within the MAPK, PI3K, and WNT pathways, genes involved in cellular migration (*MMP14*, *MMP9*, and *MTA1*), as well as anti-apoptotic proteins were downregulated. Conversely, pro-apoptotic protein expression was upregulated. Consequently, it can be deduced that quercetin effectively impedes cell cycle progression, specifically at the G2/M phase, while concurrently inhibiting migration and proliferation processes. Moreover, it induces apoptosis by suppressing MAPK-, PI3K-, and WNT-associated signaling pathways (39).

**Table 3:** Types and mechanisms of cancer treatment with quercetin.

Ingredient	Cancer	Mechanism	Phenotype	References
Quercetin	Colorectal cancer	Cell cycle stagnation in S phase; induce apoptosis.	Apoptosis	(30)
	Liver cancer	Activate the autophagy and autophagy flow; decrease the protein levels of p-AKT, mTOR, p70S6K and 4EBP1; the protein levels of p-JNK, ERK1/2 and p38MAPK are increased; inhibit proliferation; induce apoptosis.	Apoptosis Proliferation	(31)
	Pancreatic cancer	Down-regulate the expression of c-Myc and inhibite cell proliferation; decrease the level of TGF-β1 and inhibite epithelial interstitial transformation, thereby inhibiting cell migration and invasion; induce apoptosis.	Proliferation Apoptosis Migration Invasion	(32)
	Gastric cancer	Increase phosphorylation levels of p38, JNK and ERK; increase Caspase-3 activity; induce apoptosis.	Apoptosis	(33)
	Esophageal carcinoma	Inhibit proliferation; promote apoptosis.	Proliferation Apoptosis	(34)
	Ovarian cancer	Inhibit cell proliferation; induce cell apoptosis; the cell cycle is stuck in the G1 phase.	Proliferation Apoptosis	(35)
	Prostatic cancer	Reduce cell viability and induce apoptosis.	Apoptosis	(36)
	Breast cancer	Inhibit cell proliferation and cell cycle arrest in sub-G1 phase; the levels of *p53* and *p21* are up-regulated, and the expression levels of *VEGF* are down-regulated.	Proliferation Apoptosis	(37)
	Lung cancer	Inhibit cell proliferation and induce cell apoptosis; autophagy is induced.	Apoptosis	(38)
	Cervical cancer	Decrease cell viability; cell cycle arrest occurred in G2/M phase; inhibit cell migration; increase Caspase 3 activity; induce cell apoptosis.	Proliferation Migration Apoptosis	(39)

### 4.3 Gallic acid

Gallic acid typically appears as white or yellowish needle-like crystals, exhibiting solubility in water and ethanol. It possesses a diverse range of physiological activities, including antioxidant, antibacterial, and anti-tumor properties (Table 4). Gallic acid has been found to inhibit the proliferation of TE-1 cells derived from human esophageal cancer by impeding their migration and colony-forming ability while promoting apoptosis. This effect is accompanied by an elevation in ROS levels and up-regulation of pro-apoptotic proteins Caspase-3, Caspase-9, and Bax. Conversely, the expression of anti-apoptotic protein Bcl-2 along with cyclin D1 and cyclin D3 were down-regulated (40). Furthermore, gallic acid demonstrates inhibitory effects on HCT-116 and HT29 cells through its ability to suppress SRC and EGFR phosphorylation. Consequently, this inhibition leads to reduced proliferation of colon cancer cells, along with the induction of cell apoptosis (41). It was observed that gallic acid exerted a significant inhibitory effect on the migration of AGS cells, potentially mediated in part through modulation of the Ras/PI3K/AKT signaling pathway (42). Gallic acid was found to induce apoptosis in MIA PaCa-2 cells via activation of the mitochondrial signaling pathway, involving the participation of Bcl-2 and Bax proteins. Treatment with gallic acid resulted in the down-regulation of Bcl-2 protein expression while up-regulating the expression of Bax protein (43). In studies related to ovarian cancer cells, gallic acid demonstrated its ability to arrest cell cycle progression at S/G2 phase by reducing levels of cell cycle-related proteins CDC2, p-Cdc2, and cyclin B. Additionally, it activated an intrinsic apoptotic pathway mediated by Caspase-3 through upregulation of p53 (44). Lin et al. (45) showed that gallic acid exerts its apoptotic and anti-proliferative effects by inhibiting the PI3K/AKT/EGFR pathway while activating the MAPK signaling pathway. This process is accompanied by a reduction in MMP levels and an increase in ROS production, suggesting that apoptosis may be mediated through the mitochondrial apoptotic pathway and induce oxidative stress within cells. In bladder cancer studies, gallic acid has been shown to modulate cell proliferation via the PI3K/AKT and MAPK/ERK pathways, as well as inhibit bladder cancer cell invasion and migration through regulation of p-AKT/MMP2 signaling (46). BING ZHAO and MENGCAI HU (47) demonstrated in their study on cervical cancer cells that gallic acid exhibits inhibitory effects on the expression of ADAM17, EGFR, p-AKT, and p-ERK, thereby effectively impeding the progression of cervical cancer. In a separate investigation focusing on non-small-cell lung cancer, gallic acid was found to dose-dependently suppress cell proliferation. Additionally, gallic acid exhibited its regulatory potential by inducing up-regulation of p53 expression through inhibition of the PI3K/AKT pathway. This mechanism consequently modulates the expression levels of cell cycle-related proteins as well as endogenous apoptotic proteins (48). In the investigation of gallic acid’s impact on the migratory capacity of nasopharyngeal carcinoma cells, it primarily diminishes the expression of two crucial transcription factors, *AP-1* and *ETS-1*, within the *MMP1* promoter by inhibiting the p38 MAPK signaling pathway. Additionally, upregulating *TIMP-1* expression can further impede *MMP1* expression, thereby restraining tumor invasion (49). In a study conducted by Kaur et al. (50), gallic acid exhibited potential for reducing prostate cancer cell activity and inducing apoptosis; however, this effect was not observed in normal PWR-1E cells. Subsequently, researchers performed xenotransplantation experiments using animal models to validate gallic acid’s anticancer effects *in vivo*. Gallic acid exhibits anti-tumor effects on brain gliomas by inhibiting the expression of ADAM17, p-AKT, and p-ERK, thereby suppressing the PI3K/Akt and Ras/MAPK signaling pathways to mitigate tumor cell aggressiveness (51). In osteosarcoma cells, galic acid down-regulates lncRNA H19 expression, disrupting Wnt/β-catenin signaling and impeding osteosarcoma development (52). A study investigating gallic acid’s promotion of apoptosis in oral cancer cells specifically explored its mechanism. It was found that gallic acid activates CK II, leading to BIK-BAX/BAK-mediated endoplasmic reticulum-related ROS-dependent apoptosis (53).

**Table 4:** Types and mechanisms of cancer treatment with gallic acid.

Ingredient	Cancer	Mechanism	Phenotype	References
Gallic acid	Esophageal carcinoma	Reduce survival rates; inhibit cell colony formation; inhibit migration; promote apoptosis; enhance ROS level; the expression of Bax, Caspase-3 and Caspase-9 are up-regulated, while the expression of Bcl-2, cyclin D1 and cyclin D3 are down-regulated.	Apoptosis Migration Proliferation	(40)
	Colorectal cancer	Inhibit tumor growth; promote tumor apoptosis; the levels of p-SRC, p-EGFR, p-STAT3 and p-AKT are down-regulated.	Proliferation Apoptosis	(41)
	Gastric cancer	Inhibit cell viability and migration; Inhibit the expression of MMP-2/9; down-regulated the expression of PI3K, AKT-1 and p-AKT.	Migration	(42)
	Pancreatic cancer	Inhibit proliferation; promote apoptosis; decrease Δφm; up-regulated the expression of Bax and down-regulated the expression of Bcl-2.	Proliferation Apoptosis	(43)
	Ovarian cancer	Inhibit cell viability; promote cell apoptosis; the activity of Caspase-3/7 are increased, and the levels of cleaved-Caspase-3, Bad, Bax and p53 are up-regulated; the cell cycle is blocked in the S/G2 phase.	Apoptosis	(44)
	Breast cancer	Inhibit cell viability; cell cycle S phase arrest; induce cell apoptosis; reduce MMP level; promote ROS generation.	Proliferation Apoptosis	(45)
	Bladder cancer	Inhibit cell proliferation and fatty acid synthesis; cell cycle G2/M phase arrest; inhibit cell migration and invasion.	Proliferation Migration Invasion	(46)
	Cervical cancer	Decrease cell viability; inhibit cell proliferation, migration, invasion, and angiogenesis.	Proliferation Invasion	(47)
	Lung cancer	Inhibit cell viability; induce cell cycle arrest and apoptosis.	Apoptosis	(48)
	Nasopharyngeal carcinoma	Inhibit cell migration and matrix invasion; the expression of *MMP-1*, *AP-1* and *Est-1* are down-regulated, and the expression of *TIMP*-1 is increased.	Invasion	(49)
	Prostatic cancer	Decrease cell viability; induce cell apoptosis; antiangiogenesis.	Proliferation Apoptosis	(50)
	Brain glioma	Decrease cell viability; inhibit cell proliferation, migration, invasion and the formation of rat brain endothelial cell tube.	Proliferation Migration Invasion	(51)
	Osteosarcoma	Inhibit cell viability; induce cell apoptosis and cycle arrest; inhibit cell invasion and migration.	Proliferation Apoptosis Invasion Migration	(52)
	Oral cancer	Induce cell apoptosis.	Apoptosis	(53)

### 4.4 Protocatechuic acid

Protocatechuic acid, a gray-to-brown solid crystalline powder commonly found in Chinese herbs and foods, has been extensively studied for its potential anti-tumor effects. Notably, it has demonstrated the ability to induce apoptosis in tumor cells and inhibit cell proliferation across various tissues (Table 5) (60). In a study conducted by Punvittayagul et al. (54), protocatechuic acid exhibited anticancer properties in rats with diethylnitrosamine-induced hepatocarcinoma by effectively suppressing inflammation, proliferation, and promoting apoptosis. Furthermore, protocatechuic acid was found to impede HO-1-mediated activation of p21, thereby inhibiting colorectal cancer cell viability and inducing cellular apoptosis (55). In studies pertaining to esophageal cancer, protocatechuic acid has been found to exhibit inhibitory effects on tumorigenesis and inflammatory signaling, thereby suppressing the development of N-nitrosomethylbenzylamine-induced esophageal cancer (56). Motamedi et al. (57) demonstrated that protocatechuic acid effectively impedes colony formation in AGS cells by restraining cell proliferation and promoting cell apoptosis. This effect is primarily achieved through upregulating P53 expression and downregulating Bcl-2 expression levels. Furthermore, the combination of protocatechuic acid with 5-fluorouracil enhances its anti-tumor efficacy. Additionally, protocatechuic acid exerts inhibitory actions on MMP2 expression via the RhoB/PKCε and Ras/Akt cascade pathways, leading to suppression of tumor cell migration and invasion (58). Notably, for mouse breast cancer 4 T1 cells, the anti-metastatic effect does not appear to be associated with MMP2 (59).

**Table 5:** Types and mechanisms of cancer treatment with protocatechuic acid.

Ingredient	Cancer	Mechanism	Phenotype	References
Protocatechuic acid	Liver cancer	Inhibit proliferation and induce apoptosis; the expression of *P53* and Bad are up-regulated, while the expression of *Cyclin D1*, *Bcl-xl*, *TNF-α* and *IL-1β* are down-regulated.	Apoptosis Proliferation Inflammation	(54)
	Colorectal cancer	Inhibit cell viability; increase ROS level and decrease RSH level; down-regulated HO-1 expression and up-regulated p21 expression.	Apoptosis	(55)
	Esophageal carcinoma	The expression of COX-2, iNOS, p-NF-κB, sEH and PTX3 are decreased.	Inflammation	(56)
	Gastric cancer	Inhibit cell proliferation and promote cell apoptosis; increase the expression of *P53* and decrease the expression level of Bcl-2.	Apoptosis Proliferation	(57)
		Inhibit cell migration and invasion; the mRNA expression of MMP-2 is decreased and the mRNA expression of TIMP-2 is increased.	Migration Invasion	(58)
	Melanoma	Inhibit cell invasion; increase the expression of MMP, RhoB and PKCε; down-regulated the expression of Ras and p-Akt.		
	Breast cancer	Inhibit cell migration and invasion.	Migration Invasion	(59)

## 5 Liver and radiation protection

The liver functions as the primary organ responsible for drug metabolism and susceptibility to drug-induced damage. The mechanism underlying drug-induced liver injury primarily involves the direct toxic effects of drugs and their intermediates on the liver, as well as specific reactions elicited by the body towards these drugs. According to relevant surveys, approximately 15% of anti-tumor medications are associated with drug-induced liver injury (61). Consequently, in clinical practice, hepatoprotective agents are often co-administered with anti-tumor drugs to mitigate potential hepatic harm. Furthermore, chemoradiotherapy represents a crucial therapeutic approach for malignant tumors; however, it not only eradicates tumor cells but also inflicts damage upon healthy tissue cells in patients.

### 5.1 Isorhamnetin

By downregulating the TGF-β1/Smad3 and TGF-β1/p38 MAPK pathways, isorhamnetin can decrease HSC activation and ECM formation. This confirms that isorhamnetin protects mice against CCL4-induced liver fibrosis (62). Isorhamnetin application can improve the pathological injury of mouse liver tissue, lower serum liver enzyme and pro-inflammatory factor levels, and down-regulate the levels of Bax, cleaved Caspase-3, cleaved Caspase-9, Beclin-1, and p-P38/P38 in the mouse model of acute hepatitis caused by canavin A. Isorhamnetin’s hepatoprotective impact was achieved by inhibiting autophagy and apoptosis through the P38/PPAR-α signaling pathway, as evidenced by the up-regulation of PPAR-α level (63). Isorhamnetin can prevent cell death, but the combination of arachidonic acid and iron can induce mitochondrial malfunction and result in cell death. After AMPK upstream kinase CaMKK2 was knocked down, the amount of phosphorylation of AMPK was decreased, suggesting that isorhamnetin primarily reduces mitochondrial apoptosis and oxidative stress through AMPK. Isorhamnetin is therefore thought to be a possible component in the prevention of liver disease (64). Because isorhamnetin can encourage ATM activation and the recruitment of DNA repair factor 53BP1 in irradiated cells, it can prevent the development of radioactive gastrointestinal syndrome in mice (65).

### 5.2 Quercetin

Quercetin can lessen the acute liver damage brought on by CCl4; this defense may result from quercetin’s higher antioxidant capacity (66). Through a mechanism mostly associated with the reduction of Notch1 expression, quercetin can also limit M1 macrophage recruitment, polarization, and the production of inflammatory markers, thereby reducing liver inflammation and fibrosis (67). Quercetin has been shown to lower liver function-related parameters, ameliorate hepatic pathological tissue, suppress oxidative stress and apoptosis by lowering P53 and TNF-α, and prevent liver toxicity in the dobiculoxin-induced liver injury rat model (68). In a related investigation on radiation-induced brain damage, quercetin inclusion body complexes have been shown to influence the gut microbiota through modulating the microbiota-gut-brain axis. This reduces intestinal permeability and inflammation in model mice, improving the damage caused by radiation to the brain overall (69). Radiation therapy can cause side effects in cancer patients, including oral mucositis. By increasing BMI-1, quercetin can enhance wound healing by lowering the release of inflammatory agents and reactive oxygen species (70).

### 5.3 Gallic acid

The degree of liver tissue injury in the CCL4-induced Wistar rat liver injury model slightly improved following the addition of gallic acid. Gallic acid’s hepatoprotective effects were attained by downregulating pro-inflammatory indicators, scavenging free radicals, suppressing malondialdehyde levels, and activating antioxidant enzymes (71). Additionally, gallic acid might lessen the amount of liver damage brought on by anti-tuberculosis medications, mostly through the inhibition of NF-κB to lessen liver toxicity and the activation of Nrf2 and its downstream pathway to lessen drug-triggered cytotoxicity (72). The liver tissues of the mice were examined after the x-ray radiation, followed by gallic acid intragastric administration. The findings indicated that the use of gallic acid as a prophylactic measure could boost the activity of antioxidant enzymes in the liver tissues affected by radiation, diminish the oxidative and DNA damage of liver cells, and provide a protective effect from radiation on the liver of mice (73). Furthermore, due to the heightened susceptibility of salivary acicular cells to radiation, which can cause them to become dysfunctional during radiotherapy, gallic acid can regulate TLK1/1B to counteract genotoxicity, thus increasing cell survival and aiding DNA repair to reduce radiation toxicity (74).

### 5.4 Protocatechuic acid

It was discovered that protocatechuic acid could safeguard hepatocytes from the hindrance of cell viability caused by hydrogen peroxide, eradicate ROS generated by hydrogen peroxide, and diminish the activity of Caspase-3/7 following its involvement in the oxidative stress model of human hepatocellular carcinoma cell HepG2 induced by hydrogen peroxide. It appears that protocatechuic acid can safeguard hepatocytes from oxidative stress-induced apoptosis caused by reactive oxygen species (75). The protective properties of protocatechuic acid on the liver are evident in its ability to enhance oxidative stress and tissue morphology, reduce inflammatory factor expression, and lower mTOR, LC3, and Caspase-3 levels, thereby inhibiting autophagy and apoptosis (76). The hepatorenal toxicity of methotrexate, a chemotherapy drug, poses certain limitations when applied clinically. The administration of methotrexate with protocatechuic acid resulted in a decrease in the levels of TNF-α, IL-1β, and Caspase-3 in rats, suggesting that protocatechuic acid provided hepatorenal protection through its anti-oxidation, anti-inflammatory, and anti-apoptosis properties (77).

## 6 Toxicity study

The utilization of sea buckthorn can be traced back to the mid-8th century, and despite its long history, limited research has been conducted on its potential toxicity. Yuan et al. (78) conducted chromosome aberration experiments and teratogenicity experiments on mouse spermatogonia to investigate the genotoxicity and teratogenicity of sea buckthorn fruit oil. These pivotal studies serve as crucial assessments for determining the safety profile of this medicinal substance. The findings revealed that even under high dosage administration (10 mL/kg body mass) of sea buckthorn fruit oil, neither experiment exhibited any adverse reactions associated with the use of this oil. This substantiates that sea buckthorn fruit oil does not possess genotoxic or teratogenic effects. Tang et al. (79) administered sea buckthorn seed extract orally to mice and conducted acute oral toxicity, genetic toxicity, and 30-day feeding experiments. The results demonstrated no abnormalities in any aspect of the rats. Furthermore, Ruan et al. (80) performed acute toxicity tests on rats using sea buckthorn liquid at a maximum dose (causing all deaths in mice) of 19.2 g/kg, which is equivalent to 800 times the clinical use in humans. The minimum dose (mortality rate 1/10) of 11.7 g/kg also corresponds to 488 times the clinical use, indicating minimal toxicity associated with sea buckthorn consumption. Based on these experiments, it can be concluded that sea buckthorn exhibits low toxicity and high safety when used clinically as both medicine and food products due to its homologous nature.

## 7 Application

### 7.1 Patent application

**Figure 4:**
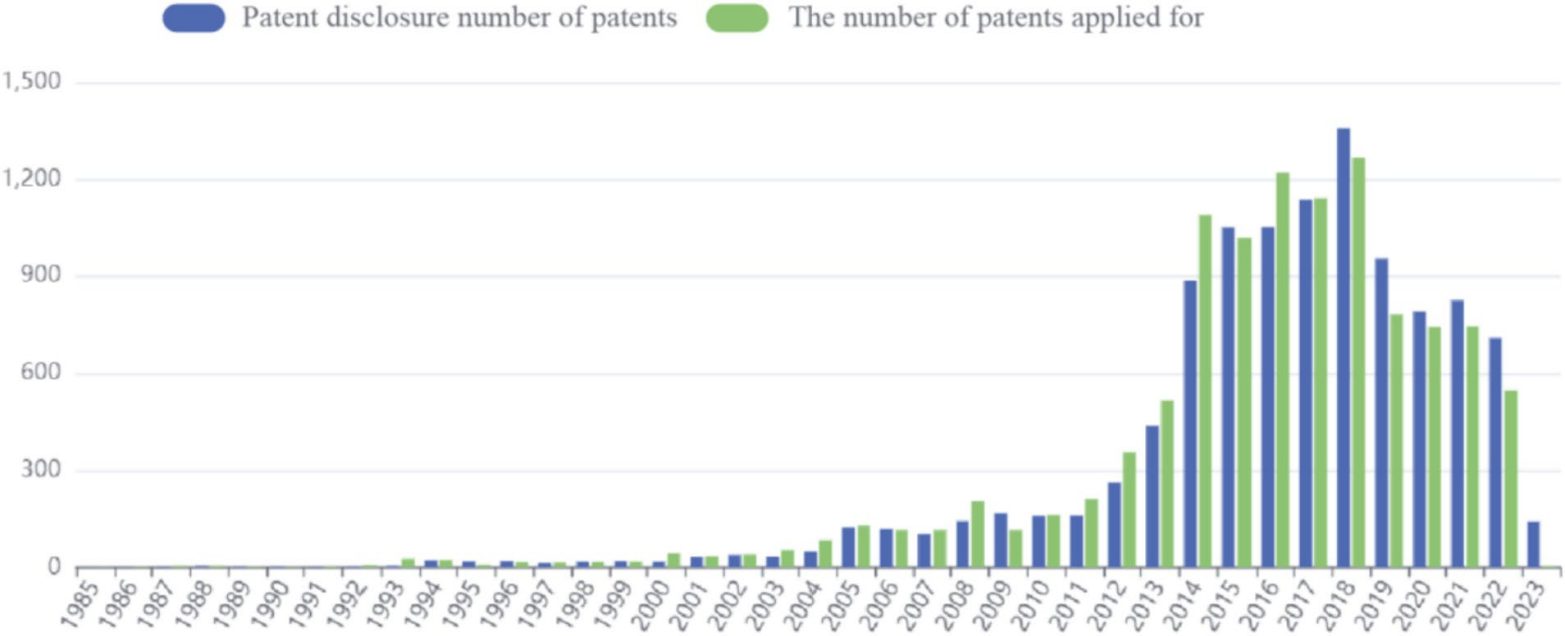
Number of sea buckthorn related patents published in recent.

Patent application data for sea buckthorn can be accessed through the Betan database.^4^ The application process commenced in 1985, and as of now (2023.4.11), a total of 10,918 patents have been published. The peak number of applications was observed in 2018, with the previous year witnessing a maximum cumulative count of 1,268 patent applications. However, in recent years, there has been a decline in the number of applications (Figure 4).

**Figure 5:**
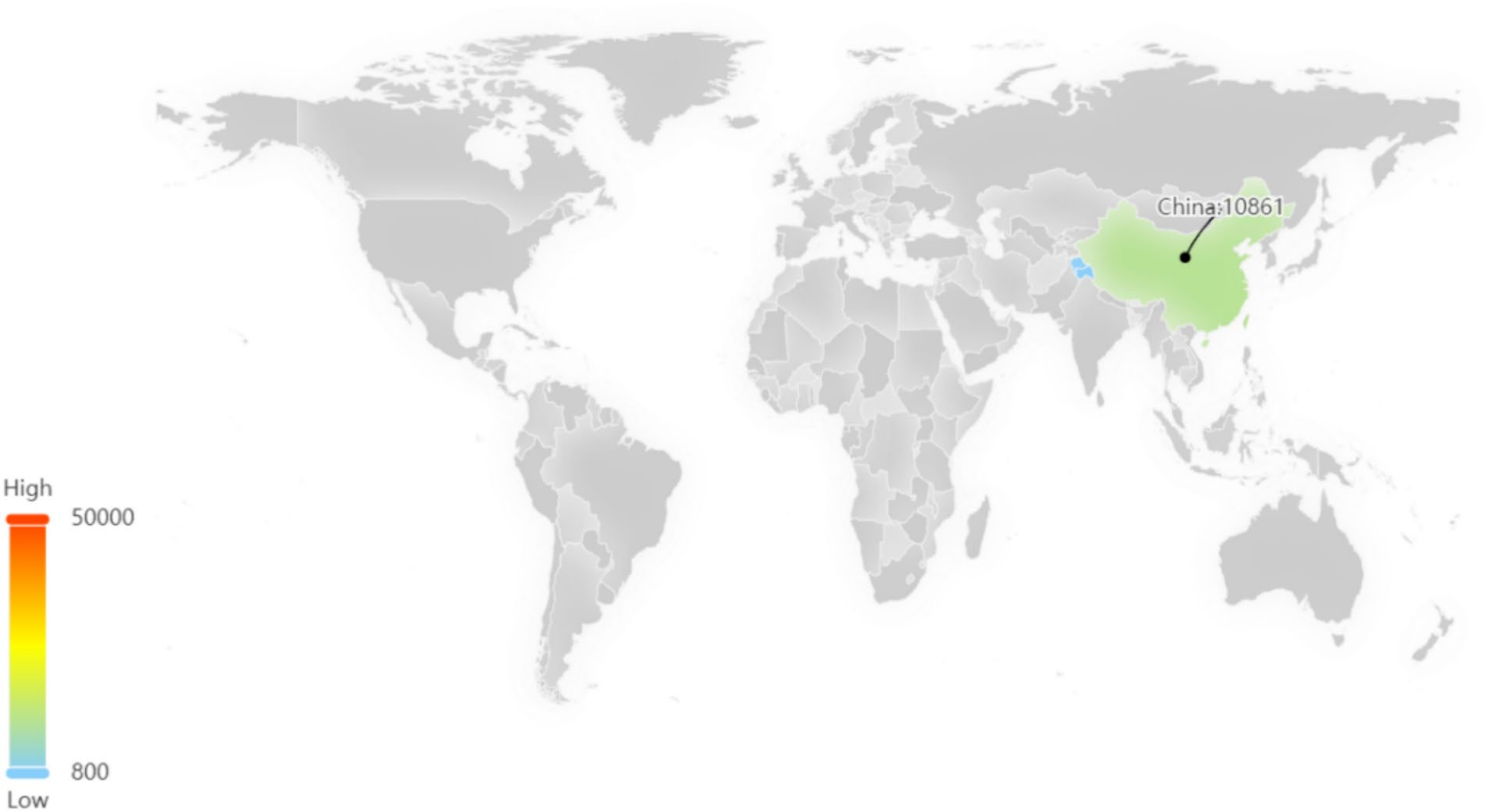
Regional distribution of sea buckthorn related patents.

Among these patents, the majority are concentrated in China, which signifies a significant level of innovation activity and intense competitive pressure within the sea buckthorn industry in China (Figure 5).

### 7.2 Food applications

Sea buckthorn, being derived from both medicine and food origins, has gained widespread utilization in food development due to its remarkable antioxidant properties, immune regulatory capabilities, and gastrointestinal protection functions. Despite the sour taste associated with sea buckthorn consumption, its flavor characteristics undergo a transformation during fermentation resulting in increased sweetness.

Liu et al. (81) conducted research on optimizing the fermentation process of sea buckthorn juice and subsequently investigated its inhibitory effects on various fungi as well as its protective effects against oxidative stress induced by H_2_O_2_. The findings demonstrated that fermented sea buckthorn juice exhibited potent antioxidant and antibacterial activities. And studies have shown that, compared with gastric cancer and colorectal cancer, sea buckthorn juice plays out in breast cancer and prostate cancer better antitumor effect (82).

Considering the declining masticatory function among the elderly population, the introduction of sea buckthorn jelly has significantly broadened the market for age-friendly food products. By utilizing various gelling agents, it becomes possible to regulate the firmness of the jelly in order to cater to individuals with diverse chewing abilities, thereby mitigating the risks associated with choking incidents and nutritional imbalances (83). Studies have shown that sea buckthorn juice addition amount of 11% sea buckthorn jelly with a higher sensory score, but due to the influence of other additives content recommendations to 9% as the best level of sea buckthorn jelly recipe (84).

Sea buckthorn leaves boast a remarkable content of polyphenols and flavonoids, which exhibit potent antibacterial, anti-inflammatory, and antioxidant properties. In analyzing the composition of sea buckthorn leaf tea, ellagic acid total content to 59.12 mg/g ranked first, as the quality control index, while ellagic acid has therapeutic effects in liver cancer, lung cancer, esophageal cancer and other cancers. After crushing sea buckthorn leaves is advantageous to the composition of precipitation (85). The chemical constituents and extracts of sea buckthorn leaf tea were investigated, revealing significant antioxidant and α-glucosidase inhibitory activities. However, heat treatment can reduce its antioxidant activity (86). After the intake of 0.1 mg/mL sea buckthorn leaf tea extracts, the DPPH radical scavenging activity is approximately 94%, ABTS radical scavenging activity in 70–90%, far higher than α-glycosidase enzyme inhibition activity. 4 mg/mL sea buckthorn leaf tea extract exhibited a moderate level of α-glucosidase inhibitory activity compared with 0.97 mg/mL (87).

The production of sea buckthorn wine has effectively addressed the issue of storage and transportation intolerance associated with sea buckthorn. Studies have demonstrated that sea buckthorn wine possesses potent free radical scavenging abilities, which gradually decline over time as it ages. The antioxidant capacity is closely linked to the vitamin C content present in sea buckthorn wine (88). After fermentation, the antioxidant activity of sea buckthorn juice increased significantly, the free radical scavenging rate increased to more than 90%, and the levels of phenolic and flavonoid active substances also increased significantly in the early stage of fermentation (89).

In addition, sea buckthorn yogurt has been found to effectively enhance the sour taste of sea buckthorn and intensify its fruit flavor. This contributes to regulating the balance of intestinal flora and boosting immunity (90). The study found that the content of VC in sea buckthorn yogurt was positively correlated with the added amount of sea buckthorn juice, but if the added amount was too high, the overall acidity of the yogurt would increase, the fermentation would be inhibited, and the protein content would be reduced. Therefore, it is recommended that the added amount of sea buckthorn juice should not exceed 15% (91).

### 7.3 Other

After undergoing processing to create various products, sea buckthorn generates a by-product known as sea buckthorn residue. Currently, the utilization of this residue primarily involves its use as animal feed or direct disposal. In order to enhance resource utilization, researchers conducted further analysis on sea buckthorn residue. A study investigating the residual fruit of sea buckthorn revealed that it retains some antioxidant properties and UPLC-Q/TOF analysis unveiled numerous compounds with free radical scavenging capabilities. Moreover, *in vitro* cell experiments demonstrated its potential to inhibit tumor cell proliferation (92). In addition, Chenyu Su et al. successfully enhanced the triterpene acid content in sea buckthorn fruit residue to optimize the overall mass fraction of triterpene acids. Subsequently, an α-glucoside inhibition experiment demonstrated that these triterpene acids effectively attenuated postprandial blood glucose levels in diabetic patients, surpassing the activity exhibited by acarbose (93). Related research on sea buckthorn fruit residue not only improves its recycling rate but also provides a basis for its further utilization.

## 8 Summary and prospect

Sea buckthorn, as a medicinal material sharing the same origin with both medicine and food, possesses significant medicinal value and holds immense potential for generating substantial economic and social benefits. This paper aims to comprehensively summarize the active constituents, anticancer properties, toxicity profile, and clinical applications of sea buckthorn. The ultimate objective is to advocate the concept of “medicine and food homology” while providing robust theoretical support for the sustainable development of sea buckthorn.

A comprehensive search was conducted on Pubmed and CNKI using the keywords “*Hippophae rhamnoides L.*,” “*Hippophae Fructus*”, “sea buckthorn,” “cancer,” “tumor” and “neoplasm” to explore recent advancements in the field of anti-tumor applications of sea buckthorn. The search yielded only two relevant reviews (94, 95). Among them, Zheng Yu et al.’s study provided limited descriptions regarding the anti-tumor effects of sea buckthorn, with a reference list primarily consisting of Chinese literature, thus diminishing its overall significance. Conversely, Beata Olas et al.’s articles presented a more comprehensive review of both *in vivo* and *in vitro* anti-tumor effects of sea buckthorn, including an insightful discussion on its potential as a radiation protective agent. However, it is worth noting that the aforementioned two reviews have a substantial historical background (2016; 2018). In this current review, we have extensively referenced numerous studies published after 2018 and meticulously summarized pertinent research on the anti-tumor effects of sea buckthorn. Furthermore, adopting the perspective of “homology of medicine and food,” we have also comprehensively examined its application in food by supplementing relevant literature cited in the previous review. Additionally, to enhance clarity and coherence, bioinformatics methods were employed to investigate the principal active components of sea buckthorn, elucidate their anticancer properties and mechanisms, and visually present our findings through informative charts.

Although the antitumor effects of sea buckthorn have been summarized, this review still exhibits several limitations. Considering the variations in active substance composition and content across different regions and varieties of sea buckthorn, as well as their corresponding therapeutic effects, it is imperative to address these factors in future research endeavors pertaining to sea buckthorn.

## Data availability statement

The original contributions presented in the study are included in the article/supplementary material, further inquiries can be directed to the corresponding author.

## Author contributions

DX: Writing – original draft, Writing – review & editing. LY: Conceptualization, Writing – original draft. FM: Writing – review & editing. DL: Formal analysis, Writing – original draft. MC: Data curation, Writing – original draft. YY: Investigation, Writing – original draft. WL: Writing – review & editing. YN: Conceptualization, Funding acquisition, Writing – original draft.

## Footnotes

^1^https://tcmsp-e.com/

^2^https://david.ncifcrf.gov/

^3^http://www.bioinformatics.com.cn/

^4^https://www.baiten.cn/

## References

[1] Chinese Pharmacopoeia. 2020th ed. Beijing: Peopleʼs Health Press (2020).

[2] Sung H Ferlay J Siegel RL Laversanne M Soerjomataram I Jemal A . Global Cancer statistics 2020: GLOBOCAN estimates of incidence and mortality worldwide for 36 cancers in 185 countries. CA Cancer J Clin. (2021) 71:209–49. doi: 10.3322/caac.21660, PMID: 33538338

[3] Wei W Zeng H Zheng R Zhang S An L Chen R . Cancer registration in China and its role in cancer prevention and control. Lancet Oncol. (2020) 21:e342–9. doi: 10.1016/S1470-2045(20)30073-5, PMID: 32615118

[4] Wang J-J Lei K-F Han F. Tumor microenvironment: recent advances in various cancer treatments. Eur Rev Med Pharmacol Sci. (2018) 22:3855–64. doi: 10.26355/eurrev_201806_15270 29949179

[5] Downer S Berkowitz SA Harlan TS Olstad DL Mozaffarian D. Food is medicine: actions to integrate food and nutrition into healthcare. BMJ. (2020) 369:m2482. doi: 10.1136/bmj.m2482, PMID: 32601089 PMC7322667

[6] Jiao L Bi L Lu Y Wang Q Gong Y Shi J . Cancer chemoprevention and therapy using chinese herbal medicine. Biol Proced Online. (2018) 20:1. doi: 10.1186/s12575-017-0066-1, PMID: 29321719 PMC5757296

[7] Zhang X Qiu H Li C Cai P Qi F. The positive role of traditional Chinese medicine as an adjunctive therapy for cancer. Biosci Trends. (2021) 15:283–98. doi: 10.5582/bst.2021.01318, PMID: 34421064

[8] Tang K . Comparison of Total flavonoids content in different parts of Seabuckthorn. Heilongjiang Agric Sci. (2022):64–7.

[9] Gong G Guan Y-Y Zhang Z-L Rahman K Wang S-J Zhou S . Isorhamnetin: a review of pharmacological effects. Biomed Pharmacother. (2020) 128:110301. doi: 10.1016/j.biopha.2020.110301, PMID: 32502837

[10] Li Y Yao J Han C Yang J Chaudhry MT Wang S . Quercetin, inflammation and immunity. Nutrients. (2016) 8:167. doi: 10.3390/nu8030167 26999194 PMC4808895

[11] Zhao M Dong J Sun J. Study on extraction and purification process optimization and application of sea buckthorn flavonoids Shihezi University (2024).

[12] Criste A Urcan AC Bunea A Pripon Furtuna FR Olah NK Madden RH . Phytochemical composition and biological activity of berries and leaves from four Romanian Sea buckthorn (*Hippophae Rhamnoides* L.) varieties. Molecules. (2020) 25:1170. doi: 10.3390/molecules25051170, PMID: 32150954 PMC7179145

[13] Zheng X Yang J Yang Y. Research progress on pharmacological effects of gallic acid. Chin J Hosp Pharm. (2017) 37:94–98+102. doi: 10.13286/j.cnki.chinhosppharmacyj.2017.01.22

[14] Chook CYB Cheung YM Ma KY Leung FP Zhu H Niu QJ . Physiological concentration of protocatechuic acid directly protects vascular endothelial function against inflammation in diabetes through Akt/eNOS pathway. Front Nutr. (2023) 10:1060226. doi: 10.3389/fnut.2023.1060226, PMID: 37025617 PMC10070727

[15] Tudor C Bohn T Iddir M Dulf FV Focşan M Rugină DO . Sea buckthorn oil as a valuable source of bioaccessible Xanthophylls. Nutrients. (2019) 12:76. doi: 10.3390/nu12010076, PMID: 31892138 PMC7020026

[16] Xia C Cui X Xiang T Fan Y Shen J. Progress in the function of palmitoleic acid. China Oils Fats. (2020) 45:39–43.

[17] Chen X Liu H Liu S Wang B. Extraction and analysis of fatty acid of sea buckthorn. Acta Agric Univ Jilin. (2009) 31:628–631+636. doi: 10.13327/j.jjlau.2009.05.042

[18] Li Y Fan B Pu N Ran X Lian T Cai Y . Isorhamnetin suppresses human gastric Cancer cell proliferation through mitochondria-dependent apoptosis. Molecules. (2022) 27:5191. doi: 10.3390/molecules27165191, PMID: 36014431 PMC9415531

[19] Saud SM Young MR Jones-Hall YL Ileva L Evbuomwan MO Wise J . Chemopreventive activity of plant flavonoid isorhamnetin in colorectal cancer is mediated by oncogenic Src and β-catenin. Cancer Res. (2013) 73:5473–84. doi: 10.1158/0008-5472.CAN-13-0525 23824743 PMC3870026

[20] Zhai T Zhang X Hei Z Jin L Han C Ko AT . Isorhamnetin inhibits human gallbladder Cancer cell proliferation and metastasis via PI3K/AKT signaling pathway inactivation. Front Pharmacol. (2021) 12:628621. doi: 10.3389/fphar.2021.628621, PMID: 33679411 PMC7927673

[21] Wang J-L Quan Q Ji R Guo X-Y Zhang J-M Li X . Isorhamnetin suppresses PANC-1 pancreatic cancer cell proliferation through S phase arrest. Biomed Pharmacother. (2018) 108:925–33. doi: 10.1016/j.biopha.2018.09.105, PMID: 30372904

[22] Wang M Xu Z Cai Q Deng Y Shi W Zhou H . Isorhamnetin inhibits progression of ovarian cancer by targeting ESR1. Ann Transl Med. (2022) 10:1216. doi: 10.21037/atm-22-5064 36544694 PMC9761148

[23] Cai F Zhang Y Li J Huang S Gao R. Isorhamnetin inhibited the proliferation and metastasis of androgen-independent prostate cancer cells by targeting the mitochondrion-dependent intrinsic apoptotic and PI3K/Akt/mTOR pathway. Biosci Rep. (2020) 40:BSR20192826. doi: 10.1042/BSR20192826 32039440 PMC7080645

[24] Park C Cha H-J Choi EO Lee H Hwang-Bo H Ji SY . Isorhamnetin induces cell cycle arrest and apoptosis via reactive oxygen species-mediated AMP-activated protein kinase signaling pathway activation in human bladder Cancer cells. Cancers. (2019) 11:1494. doi: 10.3390/cancers11101494, PMID: 31590241 PMC6826535

[25] Hu S Huang L Meng L Sun H Zhang W Xu Y. Isorhamnetin inhibits cell proliferation and induces apoptosis in breast cancer via Akt and mitogen-activated protein kinase kinase signaling pathways. Mol Med Rep. (2015) 12:6745–51. doi: 10.3892/mmr.2015.4269, PMID: 26502751 PMC4626180

[26] Luo W Liu Q Jiang N Li M Shi L. Isorhamnetin inhibited migration and invasion via suppression of Akt/ERK-mediated epithelial-to-mesenchymal transition (EMT) in A549 human non-small-cell lung cancer cells. Biosci Rep. (2019) 39:BSR20190159. doi: 10.1042/BSR20190159, PMID: 31467176 PMC6753323

[27] Ye L Ma R-H Zhang X-X Thakur K Zhang J-G Khan MR . Isorhamnetin induces apoptosis and suppresses metastasis of human endometrial carcinoma Ishikawa cells via endoplasmic reticulum stress promotion and matrix Metalloproteinase-2/9 inhibition in vitro and in vivo. Foods. (2022) 11:3415. doi: 10.3390/foods11213415, PMID: 36360027 PMC9654916

[28] Duan R Liang X Chai B Zhou Y Du H Suo Y . Isorhamnetin induces melanoma cell apoptosis via the PI3K/Akt and NF-κB pathways. Biomed Res Int. (2020) 2020:1–11. doi: 10.1155/2020/1057943 PMC7225865 32461960

[29] Wei J Su H Bi Y Li J Feng L Sheng W. Anti-proliferative effect of isorhamnetin on HeLa cells through inducing G2/M cell cycle arrest. Exp Ther Med. (2018) 15:3917–23. doi: 10.3892/etm.2018.5892, PMID: 29563987 PMC5858116

[30] Yang L Liu Y Wang M Qian Y Dong X Gu H . Quercetin-induced apoptosis of HT-29 colon cancer cells via inhibition of the Akt-CSN6-Myc signaling axis. Mol Med Rep. (2016) 14:4559–66. doi: 10.3892/mmr.2016.5818, PMID: 27748879 PMC5101998

[31] Ji Y Li L Ma Y-X Li W-T Li L Zhu H-Z . Quercetin inhibits growth of hepatocellular carcinoma by apoptosis induction in part via autophagy stimulation in mice. J Nutr Biochem. (2019) 69:108–19. doi: 10.1016/j.jnutbio.2019.03.018, PMID: 31078904 PMC9659433

[32] Guo Y Tong Y Zhu H Xiao Y Guo H Shang L . Quercetin suppresses pancreatic ductal adenocarcinoma progression via inhibition of SHH and TGF-β/Smad signaling pathways. Cell Biol Toxicol. (2021) 37:479–96. doi: 10.1007/s10565-020-09562-0, PMID: 33070227

[33] Kim MC Lee HJ Lim B Ha K-T Kim SY So I . Quercetin induces apoptosis by inhibiting MAPKs and TRPM7 channels in AGS cells. Int J Mol Med. (2014) 33:1657–63. doi: 10.3892/ijmm.2014.1704, PMID: 24647664

[34] Liao Y Sun Z Li C Zha H. Influence of quercetin on esophagus cancer Eca109 cell's proliferation and apoptosis. J Mod Med Health. (2015) 31:1458–60.

[35] Ren M-X Deng X-H Ai F Yuan G-Y Song H-Y. Effect of quercetin on the proliferation of the human ovarian cancer cell line SKOV-3 in vitro. Exp Ther Med. (2015) 10:579–83. doi: 10.3892/etm.2015.2536, PMID: 26622357 PMC4508991

[36] Ward AB Mir H Kapur N Gales DN Carriere PP Singh S. Quercetin inhibits prostate cancer by attenuating cell survival and inhibiting anti-apoptotic pathways. World J Surg Oncol. (2018) 16:108. doi: 10.1186/s12957-018-1400-z, PMID: 29898731 PMC6001031

[37] Lee YK Park SY Kim YM Lee WS Park OJ. AMP kinase/cyclooxygenase-2 pathway regulates proliferation and apoptosis of cancer cells treated with quercetin. Exp Mol Med. (2009) 41:201–7. doi: 10.3858/emm.2009.41.3.023 19293639 PMC2679247

[38] Guo H Ding H Tang X Liang M Li S Zhang J . Quercetin induces pro-apoptotic autophagy via SIRT1/AMPK signaling pathway in human lung cancer cell lines A549 and H1299 in vitro. Thorac Cancer. (2021) 12:1415–22. doi: 10.1111/1759-7714.13925, PMID: 33709560 PMC8088950

[39] Kedhari Sundaram M Raina R Afroze N Bajbouj K Hamad M Haque S . Quercetin modulates signaling pathways and induces apoptosis in cervical cancer cells. Biosci Rep. (2019) 39:BSR20190720. doi: 10.1042/BSR20190720, PMID: 31366565 PMC6692570

[40] Wu H Bavdolla N Liu L Ren Y. Inhibitory effects of gallic acid on human esophageal cancer TE-1 cells in vitro and its mechanism. China Pharm. (2022) 33:1448–54.

[41] Lin X Wang G Liu P Han L Wang T Chen K . Gallic acid suppresses colon cancer proliferation by inhibiting SRC and EGFR phosphorylation. Exp Ther Med. (2021) 21:638. doi: 10.3892/etm.2021.10070, PMID: 33968169 PMC8097205

[42] Tian Y Shu R Luo H. Effects of gallic acid on the expression of PI3K/AKT gene and its anti-metastasis effect on gastric cancer cells. Genomics Appl Biol. (2020) 39:884–9. doi: 10.13417/j.gab.039.000884

[43] Wang Y Zhao H Wang F. Gallic acid induces apoptosis of MIA PaCa-2 cells in pancreatic cancer. Chin J Gerontol. (2013) 33:5647–9. doi: 10.13325/j.cnki.acta.nutr.sin.2014.01.014

[44] He Z Liu X Wu F Wu S Rankin GO Martinez I . Gallic acid induces S and G2 phase arrest and apoptosis in human ovarian Cancer cells in vitro. Appl Sci. (2021) 11:3807. doi: 10.3390/app11093807, PMID: 34386269 PMC8356902

[45] Lin S Qin H-Z Li Z-Y Zhu H Long L Xu L-B. Gallic acid suppresses the progression of triple-negative breast cancer HCC1806 cells via modulating PI3K/AKT/EGFR and MAPK signaling pathways. Front Pharmacol. (2022) 13:1049117. doi: 10.3389/fphar.2022.1049117, PMID: 36523491 PMC9744937

[46] Liao C-C Chen S-C Huang H-P Wang C-J. Gallic acid inhibits bladder cancer cell proliferation and migration via regulating fatty acid synthase (FAS). J Food Drug Anal. (2018) 26:620–7. doi: 10.1016/j.jfda.2017.06.006, PMID: 29567231 PMC9322229

[47] Zhao B Hu M. Gallic acid reduces cell viability, proliferation, invasion and angiogenesis in human cervical cancer cells. Oncol Lett. (2013) 6:1749–55. doi: 10.3892/ol.2013.1632, PMID: 24843386 PMC4023842

[48] Ko E-B Jang Y-G Kim C-W Go R-E Lee HK Choi K-C. Gallic acid hindered lung Cancer progression by inducing cell cycle arrest and apoptosis in A549 lung Cancer cells via PI3K/Akt pathway. Biomol Ther. (2022) 30:151–61. doi: 10.4062/biomolther.2021.074, PMID: 34261818 PMC8902450

[49] Pang J-HS Yen J-H Wu H-T Huang S-T. Gallic acid inhibited matrix invasion and AP-1/ETS-1-mediated MMP-1 transcription in human nasopharyngeal carcinoma cells. Int J Mol Sci. (2017) 18:1354. doi: 10.3390/ijms18071354, PMID: 28672814 PMC5535847

[50] Kaur M Velmurugan B Rajamanickam S Agarwal R Agarwal C. Gallic acid, an active constituent of grape seed extract, exhibits anti-proliferative, pro-apoptotic and anti-tumorigenic effects against prostate carcinoma xenograft growth in nude mice. Pharm Res. (2009) 26:2133–40. doi: 10.1007/s11095-009-9926-y, PMID: 19543955 PMC2741017

[51] Lu Y Jiang F Jiang H Wu K Zheng X Cai Y . Gallic acid suppresses cell viability, proliferation, invasion and angiogenesis in human glioma cells. Eur J Pharmacol. (2010) 641:102–7. doi: 10.1016/j.ejphar.2010.05.043, PMID: 20553913 PMC3003697

[52] Pang F Ding S Li N Li Z Tian N Shi C . Gallic acid mediates tumor-suppressive effects on osteosarcoma through the H19-Wnt/β-catenin regulatory axis. J Orthop Translat. (2023) 39:34–42. doi: 10.1016/j.jot.2022.12.003, PMID: 36636358 PMC9826808

[53] Lin M-L Chen S-S. Activation of casein kinase II by Gallic acid induces BIK-BAX/BAK-mediated ER ca++-ROS-dependent apoptosis of human Oral Cancer cells. Front Physiol. (2017) 8:761. doi: 10.3389/fphys.2017.00761, PMID: 29033852 PMC5627504

[54] Punvittayagul C Luangsuphabool T Wongpoomchai R. Protocatechuic acid as a potent anticarcinogenic compound in purple rice bran against diethylnitrosamine-initiated rat hepatocarcinogenesis. Sci Rep. (2022) 12:10548. doi: 10.1038/s41598-022-14888-2, PMID: 35732709 PMC9217852

[55] Acquaviva R Tomasello B Di Giacomo C Santangelo R La Mantia A Naletova I . Protocatechuic acid, a simple plant secondary metabolite, induced apoptosis by promoting oxidative stress through HO-1 downregulation and p21 upregulation in Colon Cancer cells. Biomol Ther. (2021) 11:1485. doi: 10.3390/biom11101485, PMID: 34680118 PMC8533287

[56] Peiffer DS Zimmerman NP Wang L-S Ransom B Carmella SG Kuo C-T . Chemoprevention of esophageal cancer with black raspberries, their component anthocyanins, and a major anthocyanin metabolite, protocatechuic acid. Cancer Prev Res (Phila). (2014) 7:574–84. doi: 10.1158/1940-6207.CAPR-14-0003, PMID: 24667581 PMC6108893

[57] Motamedi Z Amini SA Raeisi E Lemoigne Y Heidarian E. Combined effects of Protocatechuic acid and 5-fluorouracil on p53 gene expression and apoptosis in gastric adenocarcinoma cells. Turk J Pharm Sci. (2020) 17:578–85. doi: 10.4274/tjps.galenos.2019.69335 33389946 PMC7786068

[58] Lin H-H Chen J-H Chou F-P Wang C-J. Protocatechuic acid inhibits cancer cell metastasis involving the down-regulation of Ras/Akt/NF-κB pathway and MMP-2 production by targeting RhoB activation. Br J Pharmacol. (2011) 162:237–54. doi: 10.1111/j.1476-5381.2010.01022.x, PMID: 20840540 PMC3012419

[59] Wang Y Gao Y Li Z Wang D Ling W. Anti-proliferative and anti-metastatic effects of protocatechuic acid on mouse breast cancer cell line 4T1. Acta Nutr Sin. (2014) 36:53–7.

[60] Kakkar S Bais S. A review on Protocatechuic acid and its pharmacological potential. ISRN Pharmacol. (2014) 2014:952943–9. doi: 10.1155/2014/952943, PMID: 25006494 PMC4005030

[61] Li L Jiang W Wang J. Clinical analysis of 275 cases of acute drug-induced liver disease. Front Med China. (2007) 1:58–61. doi: 10.1007/s11684-007-0012-8, PMID: 24557619

[62] Liu N Feng J Lu X Yao Z Liu Q Lv Y . Isorhamnetin inhibits liver fibrosis by reducing autophagy and inhibiting extracellular matrix formation via the TGF-β1/Smad3 and TGF-β1/p38 MAPK pathways. Mediat Inflamm. (2019) 2019:6175091–14. doi: 10.1155/2019/6175091, PMID: 31467486 PMC6701280

[63] Lu X Liu T Chen K Xia Y Dai W Xu S . Isorhamnetin: a hepatoprotective flavonoid inhibits apoptosis and autophagy via P38/PPAR-α pathway in mice. Biomed Pharmacother. (2018) 103:800–11. doi: 10.1016/j.biopha.2018.04.016, PMID: 29684859

[64] Dong G-Z Lee J-H Ki SH Yang JH Cho IJ Kang SH . AMPK activation by isorhamnetin protects hepatocytes against oxidative stress and mitochondrial dysfunction. Eur J Pharmacol. (2014) 740:634–40. doi: 10.1016/j.ejphar.2014.06.017, PMID: 24972246

[65] Nishiyama Y Morita A Tatsuta S Kanamaru M Sakaue M Ueda K . Isorhamnetin promotes 53BP1 recruitment through the enhancement of ATM phosphorylation and protects mice from radiation gastrointestinal syndrome. Genes. (2021) 12:1514. doi: 10.3390/genes12101514, PMID: 34680909 PMC8535534

[66] Zhang J Shi L Xu X Huang S Lu B Ji L . Therapeutic detoxification of quercetin against carbon tetrachloride-induced acute liver injury in mice and its mechanism. J Zhejiang Univ Sci B. (2014) 15:1039–47. doi: 10.1631/jzus.B1400104, PMID: 25471833 PMC4265558

[67] Li X Jin Q Yao Q Xu B Li L Zhang S . The flavonoid quercetin ameliorates liver inflammation and fibrosis by regulating hepatic macrophages activation and polarization in mice. Front Pharmacol. (2018) 9:72. doi: 10.3389/fphar.2018.00072, PMID: 29497376 PMC5819566

[68] Ahmed OM Elkomy MH Fahim HI Ashour MB Naguib IA Alghamdi BS . Rutin and quercetin counter doxorubicin-induced liver toxicity in Wistar rats via their modulatory effects on inflammation, oxidative stress, apoptosis, and Nrf2. Oxidative Med Cell Longev. (2022) 2022:2710607–19. doi: 10.1155/2022/2710607, PMID: 35936216 PMC9348941

[69] Hu J Jiao W Tang Z Wang C Li Q Wei M . Quercetin inclusion complex gels ameliorate radiation-induced brain injury by regulating gut microbiota. Biomed Pharmacother. (2023) 158:114142. doi: 10.1016/j.biopha.2022.114142, PMID: 36527844

[70] Zhang J Hong Y Liuyang Z Li H Jiang Z Tao J . Quercetin prevents radiation-induced Oral Mucositis by upregulating BMI-1. Oxidative Med Cell Longev. (2021) 2021:1–16. doi: 10.1155/2021/2231680 PMC8643266 34873428

[71] Ojeaburu SI Oriakhi K. Hepatoprotective, antioxidant and, anti-inflammatory potentials of gallic acid in carbon tetrachloride-induced hepatic damage in Wistar rats. Toxicol Rep. (2021) 8:177–85. doi: 10.1016/j.toxrep.2021.01.001, PMID: 33489777 PMC7806503

[72] Sanjay S Girish C Toi PC Bobby Z. Gallic acid attenuates isoniazid and rifampicin-induced liver injury by improving hepatic redox homeostasis through influence on Nrf2 and NF-κB signalling cascades in Wistar rats. J Pharm Pharmacol. (2021) 73:473–86. doi: 10.1093/jpp/rgaa048, PMID: 33793834

[73] Nair GG Nair CKK. Radioprotective effects of gallic acid in mice. Biomed Res Int. (2013) 2013:953079. doi: 10.1155/2013/953079, PMID: 24069607 PMC3771270

[74] Timiri Shanmugam PS Nair RP De Benedetti A Caldito G Abreo F Sunavala-Dossabhoy G. Tousled kinase activator, gallic acid, promotes homologous recombinational repair and suppresses radiation cytotoxicity in salivary gland cells. Free Radic Biol Med. (2016) 93:217–26. doi: 10.1016/j.freeradbiomed.2015.12.029, PMID: 26855419 PMC5257199

[75] Lee W-J Lee S-H. Protocatechuic acid protects hepatocytes against hydrogen peroxide-induced oxidative stress. Curr Res Food Sci. (2022) 5:222–7. doi: 10.1016/j.crfs.2022.01.006, PMID: 35106486 PMC8789513

[76] Abdelrahman RS El-Tanbouly GS. Protocatechuic acid protects against thioacetamide-induced chronic liver injury and encephalopathy in mice via modulating mTOR, p53 and the IL-6/IL-17/IL-23 immunoinflammatory pathway. Toxicol Appl Pharmacol. (2022) 440:115931. doi: 10.1016/j.taap.2022.115931 35202709

[77] Owumi SE Ajijola IJ Agbeti OM. Hepatorenal protective effects of protocatechuic acid in rats administered with anticancer drug methotrexate. Hum Exp Toxicol. (2019) 38:1254–65. doi: 10.1177/0960327119871095 31431087

[78] Yuan J Xu X Du Y Chen X Taxitiemuer A. Experimental study on genotoxicity and teratogenicity of seabuckthorn berry oil in mice. China Prev Med. (2020) 21:540–3. doi: 10.16506/j.1009-6639.2020.05.013

[79] Tang X Tan Z Zhang Y Yang W Fan B Liu J. Toxic effects of formula Seabuckthorn seed extract. J Hyg Res. (2019) 48:504–7. doi: 10.19813/j.cnki.weishengyanjiu.2019.03.025

[80] Ruan J Huang Y Yang Y. Acute toxicity and anti-aging test of sea buckthorn. Chin J New Drugs Clin Rem. (1995):325–7.

[81] Liu X Lv M Maimaitiyiming R Chen K Tuerhong N Yang J . Development of fermented sea buckthorn (*Hippophae rhamnoides* L.) juice and investigation of its antioxidant and antimicrobial activity. Front Nutr. (2023) 10:1120748. doi: 10.3389/fnut.2023.1120748 36742432 PMC9895381

[82] Boivin D Blanchette M Barrette S Moghrabi A Béliveau R. Inhibition of Cancer cell proliferation and suppression of TNF-induced activation of NFκB by edible berry juice. Anticancer Res. (2007) 27:937–48. PMID: 17465224

[83] Kim D-S Iida F. Texture characteristics of sea buckthorn (*Hippophae rhamnoides*) jelly for the elderly based on the gelling agent. Food Secur. (2022) 11:1892. doi: 10.3390/foods11131892, PMID: 35804709 PMC9266071

[84] Niu G Zhu D Wang X Li Z Liu H. Preparation of sea buckthorn jelly. Sea Buckthorn. (2006):33–6.

[85] He Q Yang K Wu X Zhang C He C Xiao P. Phenolic compounds, antioxidant activity and sensory evaluation of sea buckthorn (*Hippophae rhamnoides* L.) leaf tea. Food Sci Nutr. (2022) 11:1212–22. doi: 10.1002/fsn3.3155 36911815 PMC10003008

[86] Ma X Moilanen J Laaksonen O Yang W Tenhu E Yang B. Phenolic compounds and antioxidant activities of tea-type infusions processed from sea buckthorn (Hippophaë rhamnoides) leaves. Food Chem. (2019) 272:1–11. doi: 10.1016/j.foodchem.2018.08.006, PMID: 30309518

[87] Wang N Wen X Gao Y Lu S Li Y Shi Y . Identification and characterization of the bioactive polyphenols and volatile compounds in sea buckthorn leaves tea together with antioxidant and α-glucosidase inhibitory activities. Front Nutr. (2022) 9:890486. doi: 10.3389/fnut.2022.890486, PMID: 35571930 PMC9100590

[88] Niu G Zhu D Wang J Wang X Wei W. Study on the scavenging effects on free radical by sea buckthorn wine. J Chin Inst Food Sci Technol. (2010) 10:36–41. doi: 10.16429/j.1009-7848.2010.01.033

[89] Xia Y Zha M Liu H Shuang Q Chen Y Yang X. Novel insight into the formation of odour—active compounds in sea buckthorn wine and distilled liquor based on GC–MS and E–nose analysis. Food Secur. (2022) 11:3273. doi: 10.3390/foods11203273, PMID: 37431024 PMC9601902

[90] Yang B Ju N Ding Y Guo R Gong M. Characterization of volatile flavors of fermented sea-buckthorn yoghurt using gas chromatography-lon mobility spectroscopy. Sci Technol Food Ind (2023) 44:308–15. doi: 10.13386/j.issn1002-0306.2022080120

[91] Lin X Ma C Yang G Wu H Liu W. Development and evaluation of Set-Type Sea buckthorn yogurt. Xinjiang Agric Sci. (2016) 53:2062–8.

[92] Dienaitė L Pukalskas A Pukalskienė M Pereira CV Matias AA Venskutonis PR. Phytochemical composition, antioxidant and Antiproliferative activities of Defatted Sea buckthorn (Hippophaë rhamnoides L.) berry pomace fractions consecutively recovered by pressurized ethanol and water. Antioxidants. (2020) 9:274. doi: 10.3390/antiox9040274 32218308 PMC7222216

[93] Su C Hu N Dong Q Wang H. Study on enrichment and characterization of triterpene acid and its α- glycosidase inhibitory activity in vitro from *Hippophae rhamnoides* fruit pomace. J Food Sci Technol. (2021) 39:101–7.

[94] Zheng Y Zhang H Yu D Yu P. Progress on anti-tumor and related lmmune research of *Hippophae rhamnoides*. Chin Med Mod Distance Educ China. (2016) 14:150–2.

[95] Olas B Skalski B Ulanowska K. The anticancer activity of sea buckthorn [Elaeagnus rhamnoides (L.) a. Nelson]. Front Pharmacol. (2018) 9:232. doi: 10.3389/fphar.2018.00232 29593547 PMC5861756

